# Complete Genome Sequence of the Freshwater Bacterium Beggiatoa leptomitoformis Strain D-401

**DOI:** 10.1128/genomeA.00311-18

**Published:** 2018-04-26

**Authors:** Alexey Fomenkov, Zhiyi Sun, Tamas Vincze, Galina Dubinina, Maria Orlova, Sergey V. Tarlachkov, Brian P. Anton, Margarita Y. Grabovich, Richard J. Roberts

**Affiliations:** a New England Biolabs, Ipswich, Massachusetts, USA; b Department of Biochemistry and Cell Physiology, Voronezh State University, Voronezh, Russia; c Federal State Institution, Federal Research Centre for Fundamentals of Biotechnology, Russian Academy of Sciences, Moscow, Russia; d All-Russian Collection of Microorganisms (VKM), G. K. Skryabin Institute of Biochemistry and Physiology of Microorganisms, Russian Academy of Sciences, Pushchino, Russia; e Branch of Shemyakin and Ovchinnikov Institute of Bioorganic Chemistry, Russian Academy of Sciences, Pushchino, Russia

## Abstract

Here, we report the complete closed genome sequence and methylome analysis of Beggiatoa leptomitoformis strain D-401 (DSM 14945, UNIQEMU 779), which is quite different from the previously described Beggiatoa leptomitoformis neotype strain D-402^T^ (DSM 14946, UNIQEM U 779) with regard to morphology and lithotrophic growth in the presence of thiosulfate.

## GENOME ANNOUNCEMENT

The taxonomy of the genus Beggiatoa is still a work in progress. Despite the fact that many morphotypes of Beggiatoa have been described previously, only two species have been validated: Beggiatoa alba and Beggiatoa leptomitoformis. Previously, we reported the genome sequence of B. leptomitoformis strain D-402^T^ (1), after which we described this strain as a representative of a new species within the genus Beggiatoa (2). Since the isolates were initially described based on the morphological criteria alone and named Beggiatoa leptomitoformis, the species name was retained but its orthography was changed based on the rules of Appendix 9 of the International Code of Nomenclature of Prokaryotes (3) and validated as Beggiatoa leptomitoformis sp. nov. with the type strain D-402^T^. Here, we report the genome sequence of a second strain, B. leptomitoformis D-401. Both strains differ in their morphology and physiology, especially their ability to grow lithotrophically in the presence of thiosulfate. While B. leptomitoformis D-402^T^ is able to accumulate elemental sulfur intracellularly, D-401 is not. Therefore, comparative genomic analysis of these strains could illuminate metabolic features associated with lithotrophic growth in Beggiatoa.

B. leptomitoformis D-401 was sequenced using the Pacific Biosciences (PacBio) RS II sequencing platform. Briefly, SMRTbell libraries were constructed from genomic DNA sheared to a size ranging from ∼10 to 20 kb using the manufacturer’s instructions. DNA quality analysis and quantification were performed using the Qubit fluorimeter (Invitrogen, Eugene, OR) and 2100 Bioanalyzer (Agilent Technology, Santa Clara, CA). One 16-kb SMRTbell library was prepared according to the 20-kb PacBio sample preparation protocol, including additional separation on a BluePippin to remove fragments less than 7 kb. One size-selected and one non-size-selected library were sequenced by using C4-P6 chemistry using 2 single-molecule real-time (SMRT) cells with 240-minute collection times. Sequencing reads were processed, mapped, and assembled with the Pacific Biosciences SMRT analysis pipeline using the HGAP3 protocol and polished using Quiver (4). A total of 1.5 Gb of sequencing data was assembled into a single closed circular genome of 4,266,286 bp with 290.26-fold coverage. The assembled sequence was annotated using the NCBI Prokaryotic Genomes Annotation Pipeline (PGAP).

Surprisingly, comparative analysis of B. leptomitoformis D-401 and D-402^T^ showed more than 99.5% identity with only a 98-genomic loci difference between them. The differences consisted primarily of insertions and deletions, with only one single nucleotide polymorphism (SNP).

Epigenetic modification at each nucleotide position was measured as kinetic variation (KV) in the nucleotide incorporation rates, and methylated motifs were deduced from the KV data (5–7). Based on the KV patterns, we identified one DNA methyltransferase recognition motif presumed to contain m4C and nine presumed to contain m6A. Three additional motifs presumed to contain m5C were previously identified in strain D-402^T^ using Tet2 treatment; while we did not repeat that analysis here, we assume the results would be similar. Matching of motifs with genes of the methyltransferases responsible for each was carried out, and the results are shown in Table 1 and deposited in REBASE (8). Both the methyltransferase genes and the observed methylated motifs were identical between the two B. leptomitoformis strains.

**TABLE 1:** Summary of DNA methyltransferase genes and their modified motifs identified in B. leptomitoformis strains D-402^T^ and D-401

Motif^a^	Gene predicted in Ble402	Gene predicted in Ble401	% detected in Ble402	% detected in Ble401	Methylation type	Restriction modification type
Assigned						
G**A**T****C	M.Ble402I	M.Ble401I	99.8	100	m6A	II
GRAGC**A**G	M.Ble402II	M.Ble401II	99.5	100	m6A	II
SA****G**C**TS	M.Ble402III	M.Ble401III	20.1	99.4	m4C	II
AC**A**YNNNNNR****T****GT	S.Ble402ORF7560P	S.Ble401ORFEP	96.4	Not detected^b^	m6A	I
CA**A**YNNNNR**T**TG	S.Ble402ORF6900P	S.Ble401ORFCP	71.8	72.45	m6A	I
C**A**GNNNNNR**T**AAT	S.Ble402ORF1460P	S.Ble401ORFTP	96.9	98.8	m6A	I
Unassigned						
CATCH**A**G			100	100	m6A	II
CGG**A**G			98.9	99.4	m6A	III
CGGTC**A**			98.2	99.2	m6A	II
DCTGG**A**TD			97.7	99.9	m6A	II
GGCTG**A**			99.6	99.9	m6A	II
GTTGN**A**G			100	100	m6A	II
****T****CG**A**			98.7	99.9	m6A	II
5mC detected with mTet2 oxidation						
GGH**C**C=G****G****N**C**C	M.Ble402ORF17485P	M.Ble401ORFQP	71.56	ND^c^	5mC	II
C**C**DGG=C**C**N**G**G	M.Ble402ORF8490P	M.Ble401ORFFP	39.55	ND	5mC	II
GG**C**CNB=G****G**C**C	M.Ble402ORF16030P	M.Ble401ORFPP	29.6	ND	5mC	II
Predicted/not detected						
RGCGCY	M.Ble402ORF12705P	M.Ble401ORFKP			5mC	II
GRCGYC	M.Ble402ORF6255P	M.Ble401ORFAP			5mC	II
GCTCCA	Ble402ORF115P	Ble401ORFRP			m6A	II
GCATGC	M.Ble402ORF1215P	M1-2.Ble401ORFSP			ND	II
AAGCTT	M.Ble402ORF12455P	M.Ble401ORFJP			ND	II
TCTAGA	M.Ble402ORF3920P	M.Ble401ORFWP			ND	II
ATGCAT	M.Ble402ORFA1P				ND	II

a Modified bases are in bold, and modified bases on an opposite strand are in bold and underlined.

b The S.Ble401ORFEP modified motif was not detected directly by SMRT pipeline motif and modification software in B. leptomitoformis strain D-401, but it was confirmed manually using PBMotStat software (T.V.).

c ND, not determined.

### Accession number(s).

The complete, closed genome sequence of the B. leptomitoformis strain D-401 is available in DDBJ/ENA/GenBank with the accession number CP018889.
